# Antiviral activity of dandelion aqueous extract against pseudorabies virus both *in vitro* and *in vivo*

**DOI:** 10.3389/fvets.2022.1090398

**Published:** 2023-01-09

**Authors:** Xiaojing Cai, Yi Shao, Zhiying Wang, Yongkang Xu, Zhiyuan Ren, Lian Fu, Yan Zhu

**Affiliations:** College of Veterinary Medicine, Northeast Agricultural University, Harbin, China

**Keywords:** dandelion, pseudorabies virus, antiviral, *in vitro*, *in vivo*

## Abstract

Pseudorabies virus (PRV) is one of the most significant pathogens of swine. In recent years, the continual emergence of novel PRV variants has caused substantial economic losses in the global pig industry. PRV can infect humans leading to symptoms of acute encephalitis with implications for public health. Thus, new measures are urgently needed to prevent PRV infection. This study evaluated the anti-PRV capability of dandelion aqueous extract (DAE) *in vitro* and *in vivo*. DAE was found to inhibit the multiplication of the PRV TJ strain in PK15 cells in a concentration-dependent manner, with a 50% inhibitory concentration (IC_50_) of 0.2559 mg/mL and a selectivity index (SI) of 14.4. DAE inhibited the adsorption and replication stages of the PRV life cycle *in vitro*, and the expression of IE180, EP0, UL29, UL44, and UL52 was inhibited in the presence of DAE. *In vivo* experiment results of mice show that a 0.5 g/kg dose of DAE injected intraperitoneally protected 28.6% of the mice from the lethal challenge; decreased the viral load in the liver, lung, brain, heart, and kidney of PRV-infected mice; and attenuated brain damage caused by PRV infection. Furthermore, DAE could also ameliorate viral infection through regulation of the levels of cytokines (IFN-γ, TNF-α, and IL-4) in PRV-infected mouse serum. These results demonstrated that DAE exhibited potent inhibitory capability against PRV infection *in vitro* and *in vivo*; DAE is therefore expected to be a candidate TCM herb for use against PRV infection.

## 1. Introduction

Pseudorabies virus (PRV) is the main causative agent of Aujeszky's disease (AD, also known as pseudorabies), which belongs to the swine herpesvirus of the Alphaherpesvirinae subfamily, is an extremely infectious and lethal swine pathogen causing severe economic losses in the global swine industry (1, 2). PRV is capable of infecting a variety of hosts, including infecting numerous mammals encompassing ruminants, carnivores, and rodents (3–6). The majority of non-porcine animal species infected with PRV will eventually die as a result of fatal PRV neurotoxicity (7). As a neurotropic virus, similar to other alphaherpesviruses, PRV can cause latent infections of the nervous system (8). Notably, PRV often serves as a vital model virus for virologists to study the cross-host transmission of viruses and their ability to cause nervous system damage (2). Vaccination is a major strategy to prevent PRV infection, and the Bartha-K61 vaccine plays a pivotal role. However, since 2011, outbreaks of pseudorabies (PR) have appeared in herds vaccinated with the Bartha-K61 vaccine in China, which is caused by emerging PRV variants (9–12); these cases imply that traditional vaccines fail to provide comprehensive protection against the novel variants. Importantly, PRV has been found to infect humans and cause central nervous system (CNS) disease, fatal encephalitis, and endophthalmitis, posing a great threat to human health (13–15). Thus, it is urgently necessary to implement new measures to control PRV infection.

Traditional Chinese medicine (TCM) herbs have a wide range of sources, are easily accessible, have relatively low toxic side effects or drug residue, and are rich in natural compounds. TCM has long been recognized as a trove of resources for the search for natural antiviral drugs and has increasingly drawn attention (16). Recent studies have revealed that many TCM herbal extracts and compounds from TCM have been shown to possess anti-PRV activity. The extracts of *Kaempferia galanga L*. were found to reduce viral DNA levels in the brain, improve tissue lesion formation, and increase the survival rates of mice infected with PRV (17). The polysaccharides of *Platycodon grandiflorus* can inhibit PRV replication by reducing autophagy induced by the virus through the Akt-mTOR signaling pathway (18). Curcumin, which is the main component of *Curcuma longa*, improves the viability of hippocampal neurons infected with PRV and provides neuroprotection against PRV infection by upregulating the BDNF/TrkB pathway (19). Many TCM herbs have been found to have anti-PRV activity, but the underlying mechanisms remain unclear (20, 21).

*Taraxacum officinale*, also called the common dandelion, belongs to the Asteraceae family and is widely distributed throughout most geographical regions of China (22). Dandelion is a traditional medicinal and edible plant and has a history of thousands of years in China; it contains not only diverse bioactive components but also high dietary fiber and protein contents, as well as a large variety of amino acids and most minerals and vitamins (23). Therapeutically, dandelion has broad-spectrum antibacterial, antioxidant, diuretic, anti-inflammatory, and tumor apoptosis-inducing properties and can treat numerous diseases, such as acute inflammatory, neoplastic, urological, and diabetic syndromes (24–26). Additionally, the dandelion extract is a common antiviral agent used in TCM and has been reported to exhibit antiviral capabilities against HIV-1, influenza virus, severe acute respiratory syndrome coronavirus 2 (SARS-CoV-2), and hepatitis B virus (27–30). However, studies on the anti-PRV capability of dandelion have not been undertaken. Therefore, in this study, we prepared a dandelion aqueous extract (DAE) by the decoction method to systematically evaluate the anti-PRV activity of DAE, aiming to develop a new TCM for preventing PRV infections.

## 2. Materials and methods

### 2.1. Cells and virus

Porcine kidney 15 (PK15) cells were cultured in Dulbecco's modified Eagle's medium (DMEM, Gibco, USA) supplemented with 10% (v/v) fetal bovine serum (FBS, Gibco, USA) and 1% penicillin/streptomycin. The medium used for cytotoxicity and antiviral tests contained 2% serum. The PRV TJ strain was isolated from infected pigs that received a commercial vaccine, which was kindly provided by the Harbin Veterinary Research Institute, Chinese Academy of Agricultural Sciences. The virus was propagated in PK15 cells, and the titer of the virus was 10^7.04^ 50% tissue culture infective dose (TCID_50_)/mL as determined by the Reed-Muench method (31).

### 2.2. Preparation of the DAE

The entire plant of *Taraxacum officinale* was collected from Linyi City, Shandong Province, China (N35°06′52", E118°32′64") in September 2021 and was authenticated by Dr. Zunlai Sheng, Northeast Agricultural University. The voucher specimen of dandelion was stored in the Herbarium of Northeast Agricultural University. The herbs were boiled in water to obtain dandelion aqueous extract (DAE). In brief, 100 g of the dried entire dandelion plant was dissolved in water for 0.5 h at room temperature, followed by extraction twice with water at 100°C for 2 h. The mixtures were pooled together and filtered through a 0.45 μm membrane to remove impurities. Then, the filtrate was concentrated by a rotary evaporator and finally dried to remove the moisture, yielding a light-yellow powder.

### 2.3. Cytotoxicity assay

The cytotoxicity of DAE was assessed by the detection of cell viability using a Cell Counting Kit 8 (CCK8, Bimake, USA) assay. Briefly, 10^4^/mL PK15 cells were seeded in 96-well plates (Costar, USA) and cultured in a 5% CO_2_ atmosphere at 37°C until the cells grew to 80–90% confluence. These cells were then treated with DAE ranging in concentration from 0.25 to 8 mg/mL for 48 h, and each concentration was replicated six times. After incubation for 48 h, the cell state was observed under an optical microscope, then the cells in each well were added to 10% (v/v) CCK8 and incubated for 1 h at 37°C. The absorbance was measured at a wavelength of 450 nm. The cell viability was presented as a percentage of cell viability compared with the control (no DAE treatment). The 50% cytotoxic concentration (CC_50_) of DAE was calculated by GraphPad Prism 8.0 software (GraphPad Software, San Diego, CA).

### 2.4. Effect of DAE on PRV-infected PK15 cells

PK15 cells were grown at 37°C and in an atmosphere of 5% CO_2_ until they reached a density of 80–90% confluence in 96-well plates. DAE (2–0.25 mg/mL) was mixed with an equal volume of PRV (100 TCID_50_) for 1 h at 37 °C, the mixture was then added to PK15 cells and incubated for 1 h for virus adsorption. After 1 h of incubation, the mixture was removed, and these cells were thoroughly washed three times with PBS and overlaid with DMEM containing 2% FBS. The cytopathic effect (CPE) was monitored daily by light microscopy, and the antiviral activity of different concentrations of DAE was determined by CCK8 assay when PK15 cells infected with PRV showed CPE up to 70–80%. In parallel, the experiments described above were performed in 6-well plates to obtain infected cells for measuring PRV gene copies by fluorescence quantitative polymerase chain reaction (FQ-PCR). The following formula is used to calculate the virus inhibition ratio: virus inhibition ratio = (absorbance value of DAE-treated group–absorbance value of PRV control group)/(absorbance value of cell control group–absorbance value of PRV control group) × 100%. Calculations were performed using the software GraphPad Prism 8.0 to determine the 50% inhibitory concentration (IC_50_). The selectivity index (SI), with the formula SI = CC_50_/IC_50_, was used to judge the safety margin of the drug effect.

### 2.5. Mode of action assays

In the virus adsorption assay, PK15 cells were cooled to 4°C in advance for 1 h and then challenged with PRV (100 TCID_50_) simultaneously with the corresponding concentrations of DAE (2, 1, or 0.5 mg/mL) for 1 h at 4°C. The unbound inoculum was carefully removed, and the cells were thoroughly washed three times in ice-cold PBS and overlaid with DMEM containing 2% FBS. The infected cells were collected after 40 h of incubation and tested for viral gene copies by FQ-PCR.

In the virus entry assay, PK15 cells were challenged with 100 TCID_50_ of PRV at 4°C for 1 h. The unbound viruses were removed, and the PK15 cells were incubated with DAE (2, 1, or 0.5 mg/mL) for 1 h at 37°C to allow entry. After thoroughly washing three times with PBS, the cells were covered with DMEM containing 2% FBS. The infected cells were collected after 40 h of incubation and tested for viral gene copies by FQ-PCR.

In the virus replication assay, PK15 cells grown as a monolayer were challenged with 100 TCID_50_ of PRV at 37°C for 1 h. After 1 h of incubation to allow virus adsorption, following the removal of unbound viruses, the cells were fully washed with PBS three times and added with the DAE-containing solutions (2, 1, or 0.5 mg/mL). The infected cells were collected after 40 h of incubation and tested for viral gene copies by FQ-PCR.

### 2.6. DNA extraction and FQ-PCR

A TIANamp Genomic DNA Kit (Tiangen Biotech, China) was used to extract the total DNA following the manufacturer's instructions. The FQ-PCR assay was used to detect the PRV gene copy number in infected cells. Purified plasmids containing the gI gene were initially used to generate standard curves. Detailed information about the probe and primer sequences can be found in the Supplementary Table 1. The reaction thermal cycling conditions of FQ-PCR were as follows: 94°C for 5 min, followed by a two-step cycle (94°C for 35 s and then 62°C for 35 s) for 40 cycles. The viral genomic DNA copy numbers were calculated by the standard curve and the CT value of the sample.

### 2.7. Gene expression analysis

The effect of DAE on PRV gene expression in PK15 cells was detected by relative quantitative PCR. In brief, PK15 cells grown in 6-well plates were challenged with 100 TCID_50_ of PRV for 1 h, the viral inoculum was then removed, and DMEM with or without DAE (2 mg/mL) was added to the cultures. The total RNA of infected cells was extracted using the RNA Easy Fast Tissue/Cell Kit (Tiangen Biotech, China), and cDNA was obtained by reverse transcription using FastKing-RT SuperMix (Tiangen Biotech, China) following the manufacturer's instructions. The relative quantitative PCR was performed with the SYBR Green qPCR Master Mix (Bimake, USA) on a Roche 480 instrument, and the primers are included in the Supplementary Table 1. The reaction thermal cycling conditions were predenaturation at 95°C for 5 min followed by a three-step cycling process (95°C for 15 s, 55°C for 30 s, and 72°C for 30 s) for 40 cycles. The relative level of RNA expression in PRV-infected cells was determined by the 2^−ΔΔCT^ method. Each reaction was performed in triplicate, and normalization was performed using the housekeeping gene β-actin.

### 2.8. Mouse challenge protection test

Thirty 5-week-old female BALB/c mice were purchased from Changsheng Biotechnology Co., Ltd. (China). The mice were randomly divided into three groups of ten mice each after acclimation for 1 week, and the specific groupings are shown in Table 1. In addition to the uninfected control group (0.1 mL saline), the mice of the infected control and DAE-treated group were intramuscularly injected with 0.1 mL saline containing 2 × 10^3^ TCID_50_ of PRV. At 1 h after artificial infection, the mice in the DAE-treated group received intraperitoneal injection of DAE at a dose of 0.5 g/kg once per day for 5 days. At the same time, the mice of the uninfected control and infected control groups received equal volumes of saline instead of DAE. The weight, behavior, and health condition of the mice were recorded daily. At 4 h after the 4th DAE treatment (3 dpi), three live mice were randomly selected from each group. Blood samples were collected under anesthetization, euthanized by cervical dislocation, and subjected to comprehensive dissection to harvest the brain, kidney, lung, liver, and heart for analysis. The number of remaining mice per day was recorded to determine the survival rate during the experimental period.

**Table 1:** Groups of animal experiment.

**Name**	**Group**	**Injection content**
A	Uninfected control	Saline
B	Infected control	PRV + saline
C	DAE-treated	PRV + DAE (0.5 g/kg)

The lung, kidney, liver, heart, and brain were harvested from each group and homogenized to extract DNA for determination of the viral load by FQ-PCR. The brains of 2 mice in each group were fixed in 4% formaldehyde, embedded in paraffin, and stained with hematoxylin-eosin (HE) solution for histopathological studies under a light microscope.

Blood samples obtained by orbital blood collection were coagulated naturally at room temperature for 30 min and then centrifuged at 3,000 r/min for 10 min. The supernatant was carefully collected for the detection of cytokines (IL-4, IFN-γ, and TNF-α) in the mouse serum, which was measured using an ELISA Kit (Nanjing JianCheng, China).

### 2.9. Statistical analysis

All experiments with cells were repeated at least three times, and the results are presented as the mean ± standard deviation (SD). GraphPad Prism 8.0 software (La Jolla, CA, USA) was used for statistical analysis. The statistical significance of the data was assessed by two-way analysis of variance (ANOVA) and one-way ANOVA with Dunnett's multiple-comparison test. A *p* < 0.05 was considered statistically significant.

## 3. Results

### 3.1. Cytotoxicity of DAE

**Figure 1:**
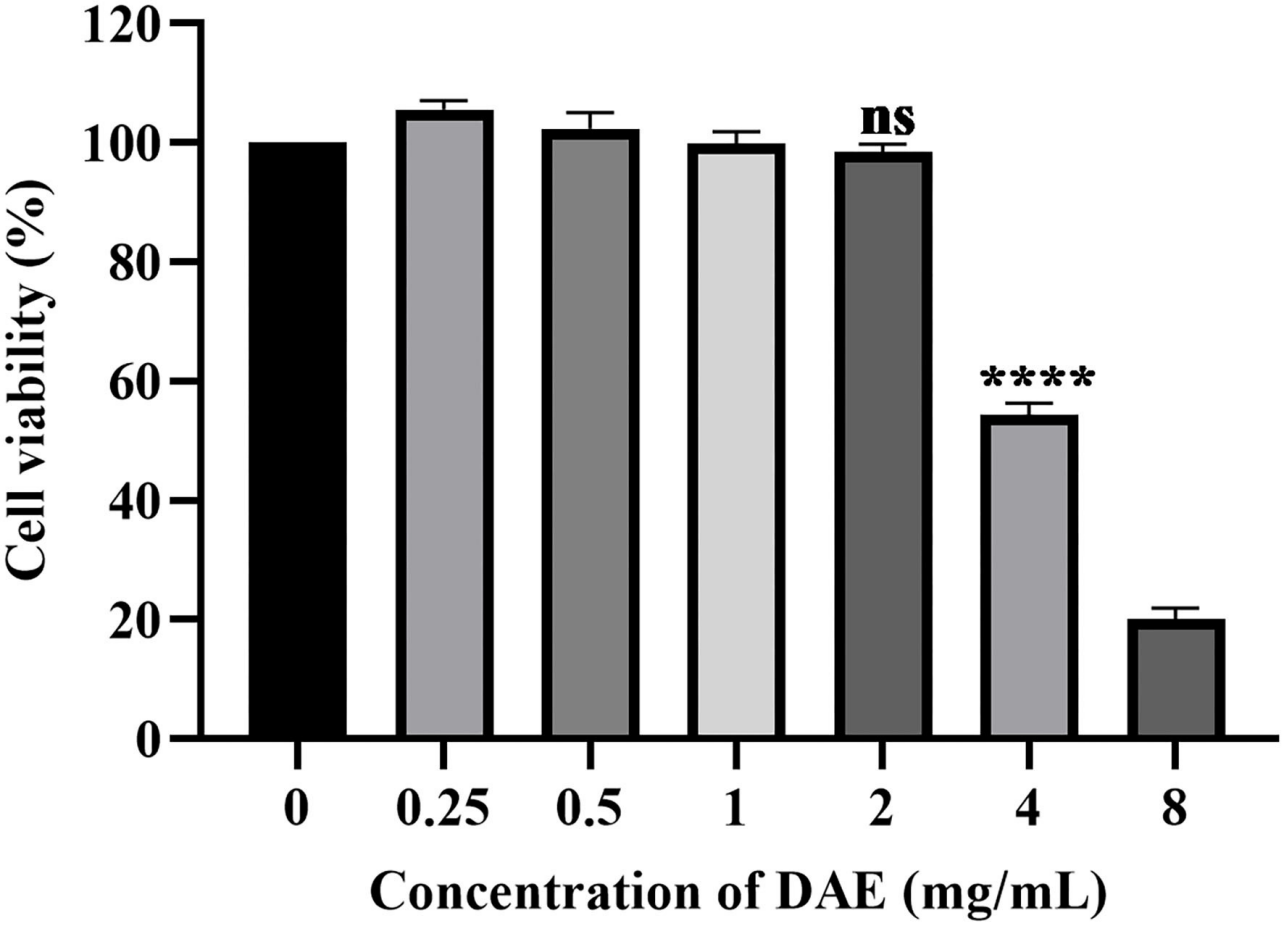
Cytotoxicity of DAE at different concentrations in PK15 cells. A CCK8 assay was used to determine the toxicity of DAE to PK15 cells at concentrations of 8, 4, 2, 1, 0.5, and 0.25 mg/mL. The cell viability was expressed as a percentage of the control cell viability (****P < 0.0001, ns: *P* > 0.05).

The dried whole dandelion plant was extracted with boiling water, yielding DAE (14.2% w/w). In this study, the cytotoxicity of DAE was determined by assessing the viability of cells by the CCK8 assay. The results are represented in Figure 1, the viability of PK15 cells decreased gradually with increasing concentrations of DAE. The viability of PK15 cells was not significantly different from that of the control cells when the concentration was no more than 2 mg/mL, so 2 mg/mL was the maximum non-toxic concentration of DAE on PK15 cells, which was therefore selected for subsequent cell experiments. The CC50 value, listed in Table 2, was 3.684 mg/mL.

**Table 2:** The CC_50_, IC_50_, and SI of the DAE.

**Substance**	**CC_50_ (mg/mL)**	**IC_50_ (mg/mL)**	**SI**
DAE	3.684 ± 0.11	0.2559 ± 0.08	14.4 ± 0.03

### 3.2. DAE inhibits PRV infection in PK15 cells

**Figure 2:**
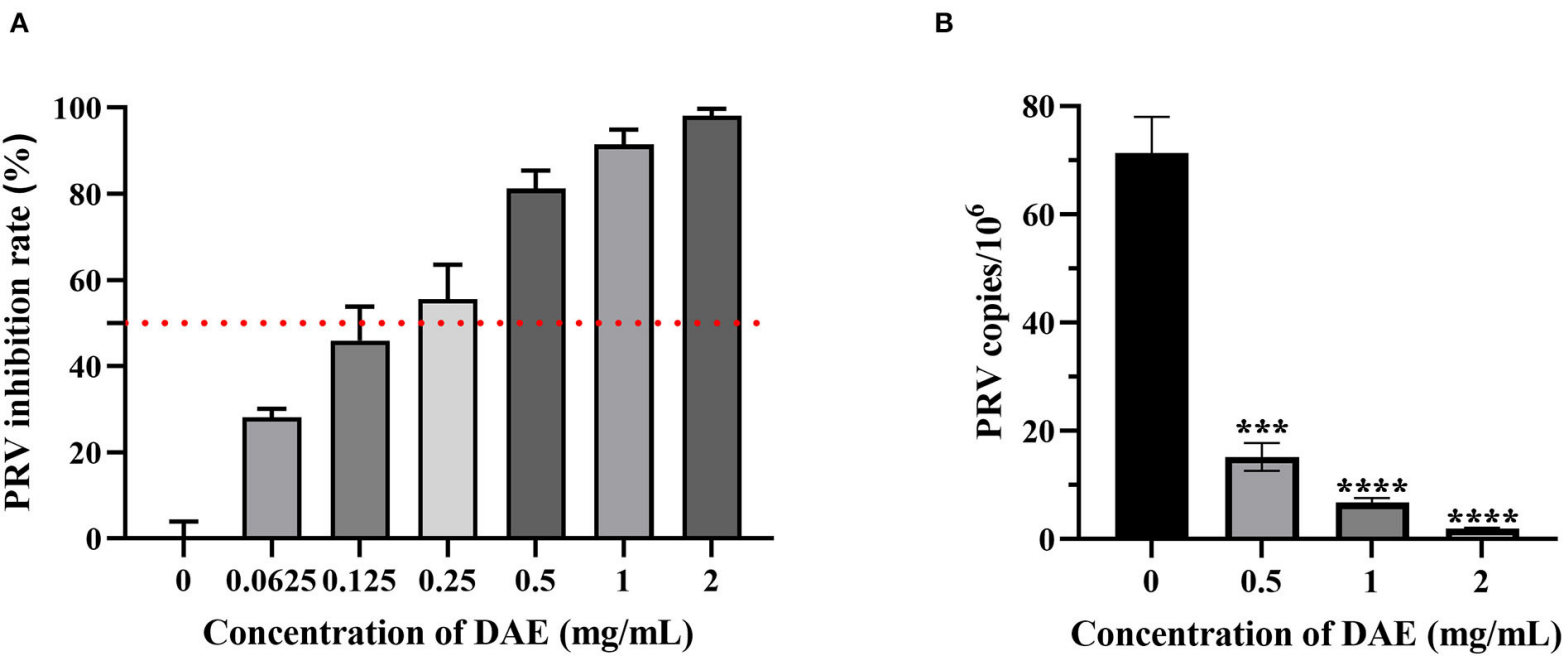
Antiviral effect of DAE on PRV in PK15 cells. **(A)** The inhibitory effect of DAE on PRV in PK15 cells. PRV (100 TCID_50_) was mixed with an equal volume of DAE (0.0625–2 mg/mL) at 37°C for 1 h, and the mixture was overlaid on PK15 cells for 1 h before replacement with 2% DMEM. After incubation for 48 h, CCK8 assays were performed, and the results are expressed as the percent inhibition of the drug-treated group compared with the untreated group. **(B)** PRV copies in infected cells were quantified by FQ-PCR (****P < 0.0001, ***P < 0.001).

A CCK8 assay and FQ-PCR were performed to explore the capability of DAE to inhibit PRV proliferation in PK15 cells, as mentioned previously. PK15 cells were challenged with PRV in the presence or absence of DAE for 1 h. The cell viability was evaluated by CCK8 assay, and the PRV gene copies of infected cells were determined by FQ-PCR. The CCK8 assay results indicated that DAE has significant inhibitory activity on PRV infection in a concentration-dependent manner. At a DAE concentration of 2 mg/mL, the highest inhibition rate was 98.07%, and at a DAE concentration of 0.25 mg/mL, more than 50% of PRV replication was still inhibited in PK15 cells (Figure 2A). The IC_50_ of DAE was estimated to be 0.2559 mg/mL, and the SI was 14.4 (Table 2). Similar to the results of the CCK8 assay, in the FQ-PCR assay, the PRV gene copy numbers were significantly reduced in the presence of DAE, in a concentration-dependent manner (Figure 2B). The viral copy numbers were decreased by 36.93, 9.58, and 3.75 times at concentrations of 2, 1, and 0.5 mg/mL, respectively. Taken together, these results indicated that DAE can significantly inhibit PRV infection in PK15 cells.

### 3.3. DAE inhibits the adsorption and replication of PRV in PK15 cells

**Figure 3:**
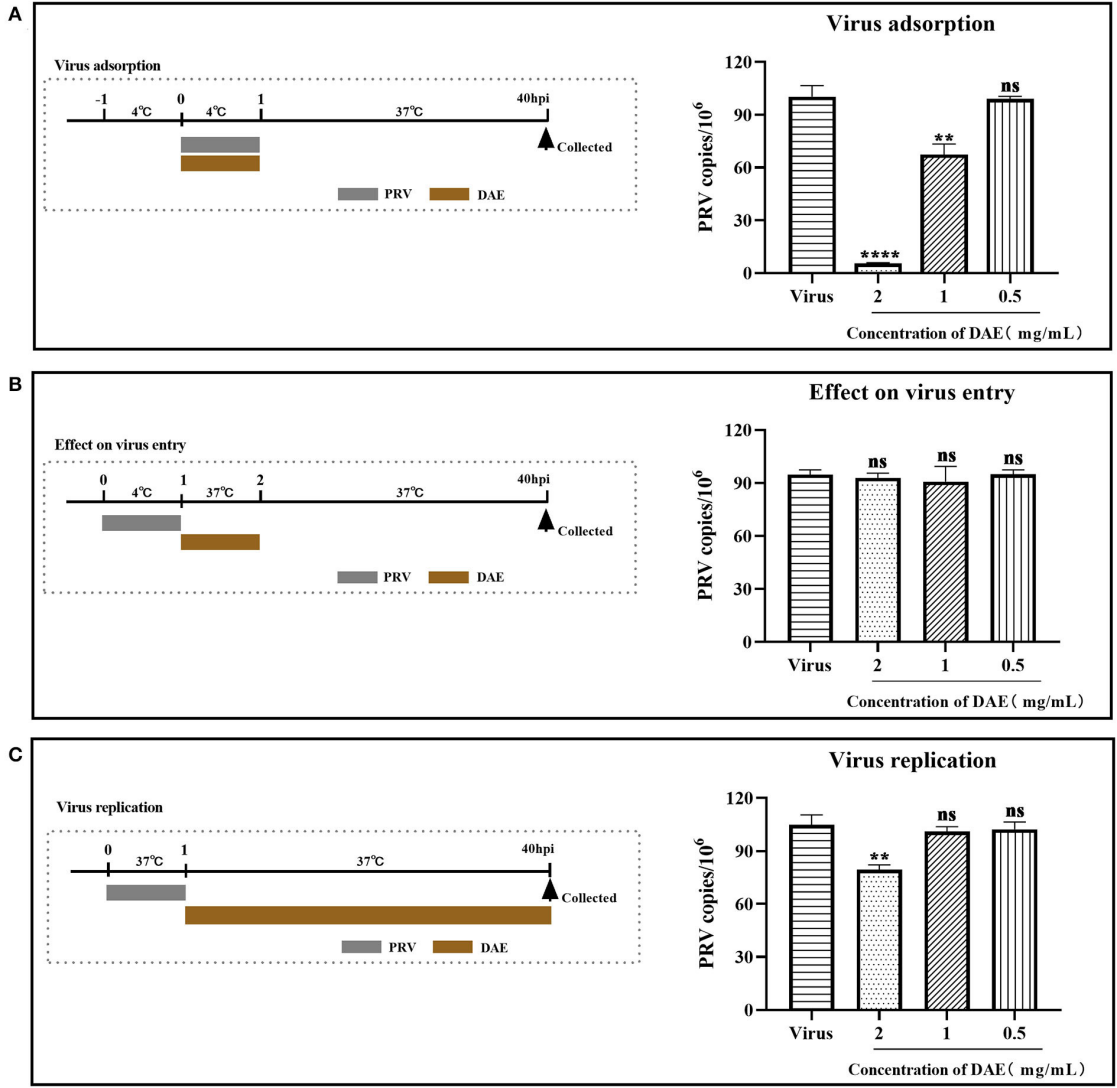
Influence of different DAE treatment conditions on PRV infection. Upon the addition of DAE under different treatment conditions, after 40 h, infected cells were collected, and the virus copy number in the PK15 cells was determined by FQ-PCR. **(A)** Virus adsorption, **(B)** effect on virus entry, **(C)** virus replication (****P < 0.0001, **P < 0.01, ns: *P* > 0.05).

To determine which stage of the PRV life cycle was influenced by DAE, a mode of action assay was conducted. Upon the addition of DAE under different treatment conditions, after 40 h, infected cells were harvested, and the PRV gene copy numbers of the infected cells were determined by FQ-PCR. For the virus adsorption and replication assays, the PRV gene copy numbers within infected cells were significantly reduced by DAE at 2 mg/mL (Figures 3A,C), especially in the adsorption assay, and DAE reduced the viral copy number by 18.1 times. However, the number of PRV gene copies was not significantly different from that in the control group in the virus entry assay (Figure 3B). The results suggested that DAE exerted antiviral effects on the viral adsorption and replication stages of PRV in PK15 cells.

### 3.4. DAE inhibits PRV gene expression

**Figure 4:**
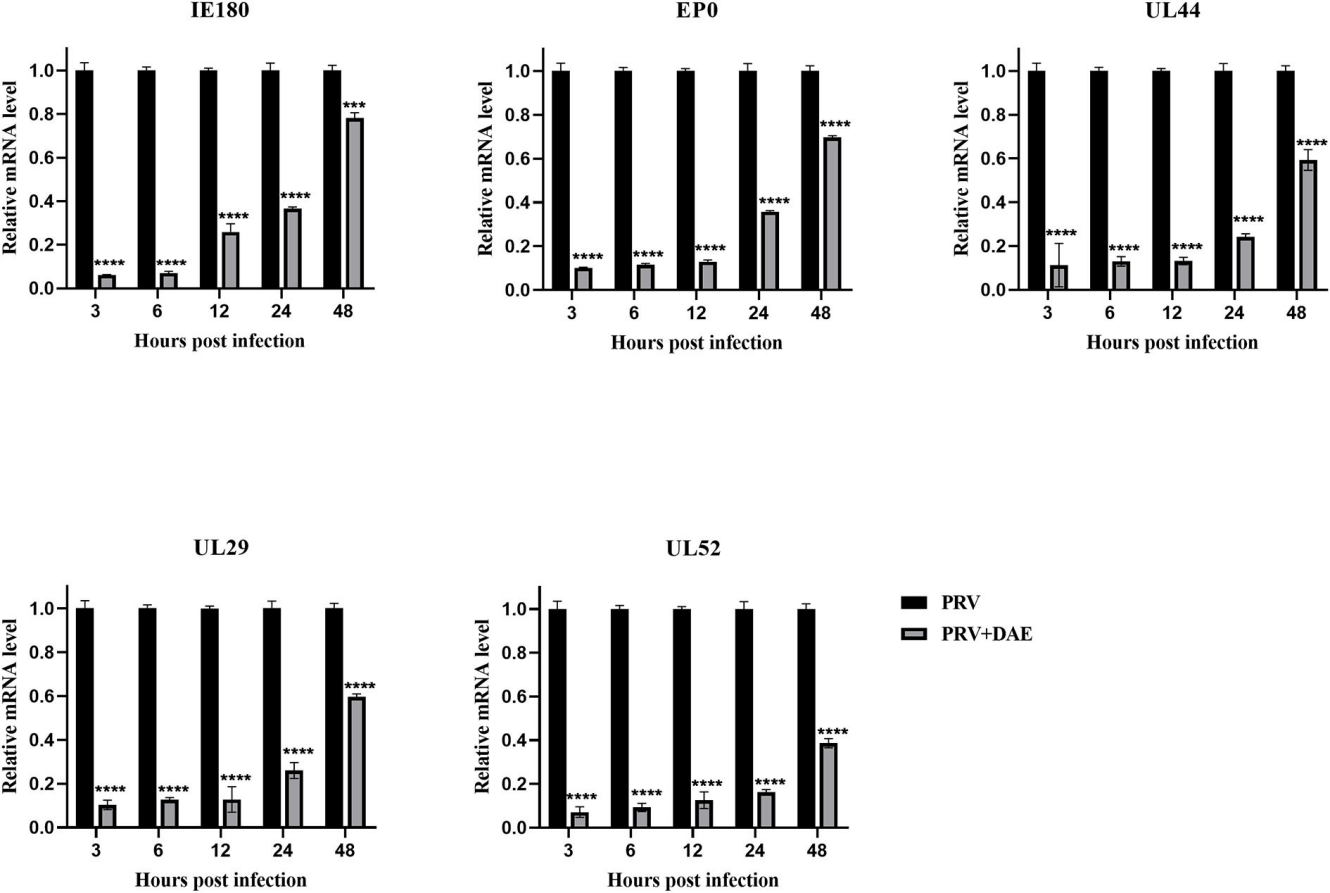
DAE suppressed the expression of PRV genes. In the presence or absence of emodin, the expression of PRV genes (IE180, EP0, UL52, UL29, and UL44) was detected by relative quantitative PCR at 3, 6, 12, 24, and 48 hpi (*n* = 3 in each group) (****P < 0.0001, ***P < 0.001).

The mode of action results showed that DAE exerted anti-PRV activity by blocking the adsorption and replication stages of the PRV life cycle. The expression of PRV genes is the initial step of viral DNA entry into the nucleus and the key step in viral replication (2). Infected cells were harvested at 3, 6, 12, 24, and 48 hpi, and the expression of the PRV immediate early gene IE180, early gene EP0, and other genes (UL52, UL29, and UL44) was measured by relative quantitative PCR to explore the effect of DAE on PRV gene expression. The relative expression levels of the tested genes are depicted in Figure 4, the relative expression of all detected genes in the DAE-treated group showed an upward trend, but compared with the virus group, DAE significantly inhibited the expression of all detected genes within 48 h at a concentration of 2 mg/mL. The results showed that the antiviral activity of DAE may be achieved by inhibiting PRV gene expression.

### 3.5. DAE inhibits PRV infection in mice

**Figure 5:**
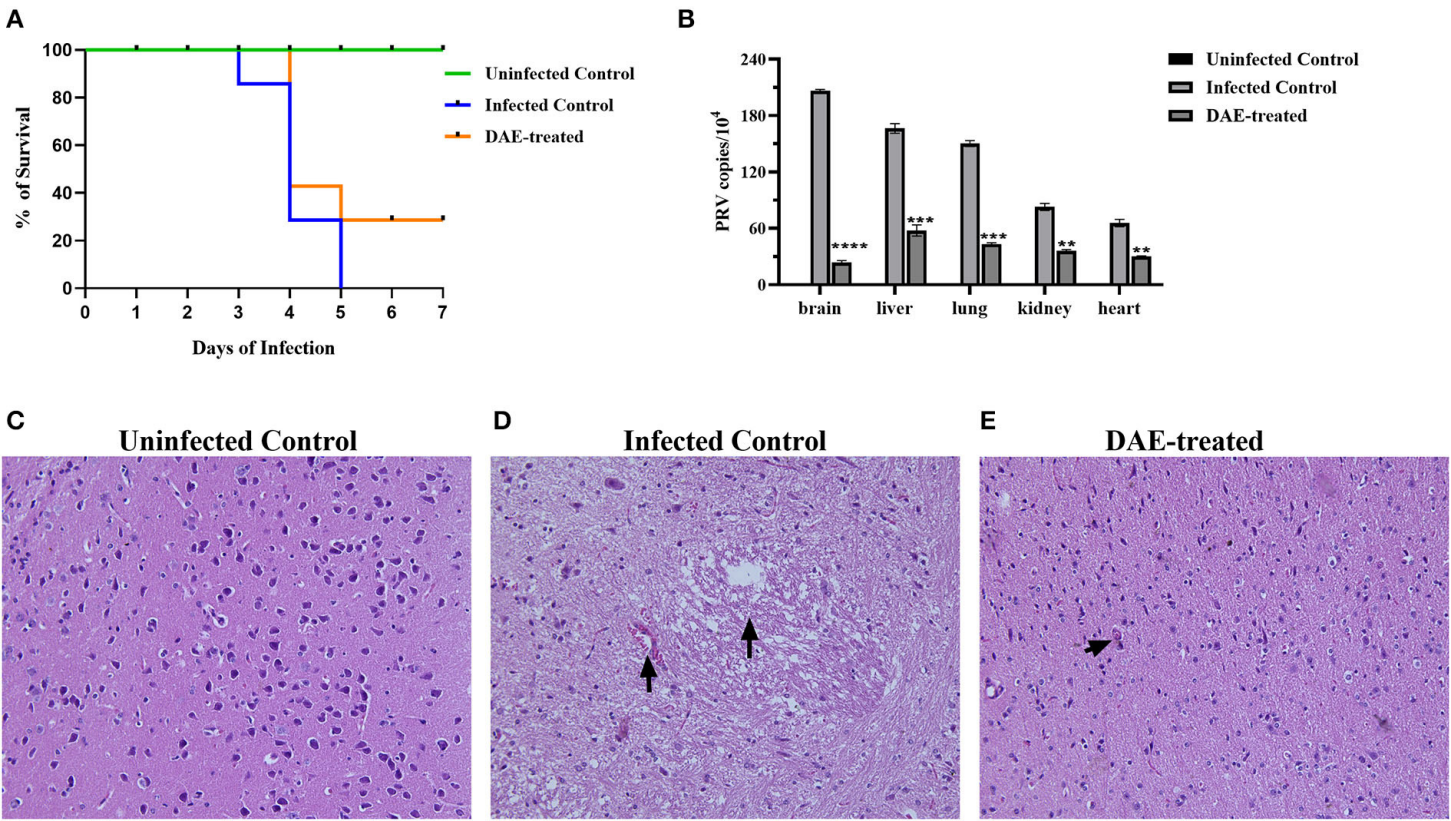
DAE showed antiviral activity against PRV in mice. **(A)** The survival rate of mice in each group. **(B)** The viral load in the heart, kidney, liver, lung, and brain of mice in each group. The viral copy number in different organs was detected by FQ-PCR 76 h after PRV challenge. Histopathological sections of mouse brain were harvested from the uninfected control **(C)**, infected control **(D)**, and DAE-treated **(E)** mice, with hematoxylin-eosin staining (****P < 0.0001, ***P < 0.001, **P < 0.01).

The antiviral effects of DAE have been shown *in vitro* in previous studies. To further explore the anti-PRV capability of DAE *in vivo*, animal experiments were performed as described above. The mice were observed daily for clinical symptoms. At 3 days post-infection (dpi), the mice from all PRV-infected groups gradually exhibited neurological symptoms, especially the infected control mice, which manifested as excitement, itching, scratching and biting of the injection site, and bleeding. As shown in Figure 5A, in the infected control group, mice began dying at 3 dpi, and the number of surviving mice was reduced to two (28.6%) at 4 dpi and 0 (0%) at 5 dpi. Comparatively, the death of the mice was delayed to the 4th day in the DAE-treated group, the number of surviving mice was two (28.6%) at 5 dpi, and two mice were still alive at 7 dpi. For the uninfected groups of mice, no deaths occurred from 1 to 7 dpi.

Viral load is one of the most vital indicators to evaluate the effectiveness of antiviral *in vivo* and is the most important parameter in the evaluation of PRV multiplication (32, 33). To evaluate the viral load in mouse organs, at 76 h after the challenge, three mice from each group were randomly selected, and their liver, kidney, lung, heart, and brain were collected. Viral DNA was extracted and quantified by FQ-PCR. The results showed that the PRV content in the mouse brain was the highest, followed by that in the liver, lung, kidney, and heart. Compared with the infected control group, the PRV content in each tested organ was significantly decreased after DAE treatment, especially in the brain, and the PRV content was reduced ~7.72 times (Figure 5B). Viral infection usually causes damage to healthy tissues. As the viral content in the brain of infected mice was the highest, we collected the brains of mice in each group for histopathological examination to further explore whether DAE has protective effects on the brain. In contrast with the brain of mice in the uninfected control group (Figure 5C), a small amount of blood stasis and neuronal degeneration and necrosis were observed in the brain of the infected control group mice (Figure 5D). The lymphocytic infiltration was less noticeable in the mouse brain of the DAE-treated group (Figure 5E). As expected, DAE reduced PRV infection in mice.

### 3.6. Changes in serum cytokine levels

**Figure 6:**
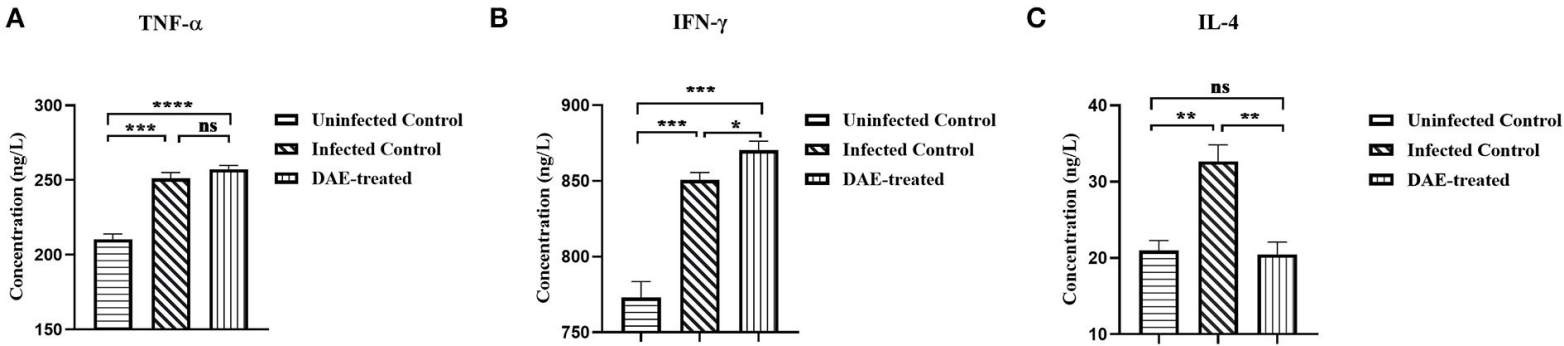
The concentrations of TNF-α **(A)**, IFN-γ **(B)**, and IL-4 **(C)** in serum obtained from test mice. At 76 h post-PRV infection, three mice from each group were randomly selected for the collection of blood, which was used to determine the levels of three cytokines in mouse serum (****P < 0.0001, ***P < 0.001, **P < 0.01, *P < 0.05, ns: *P* > 0.05).

Cytokines play a crucial role in resisting the invasion of foreign pathogens by regulating immune and inflammatory responses. As mentioned above, the immune function status of the mice was assessed by measuring cytokines with ELISA kits. The results are illustrated in Figure 6, compared with those in the uninfected control mice, the levels of TNF-α, IL-4, and IFN-γ were increased significantly in mice infected with PRV. The levels of the three tested cytokines changed differently after DAE treatment in comparison with those in the infected control, there was no significant difference in the concentration of TNF-α between the DAE-treated group and infected control group mice (Figure 6A); the concentration of IFN-γ increased significantly after DAE treatment (Figure 6B); and for IL-4, DAE treatment reduced the increased level of this cytokine caused by PRV infection to normal uninfected control levels (Figure 6C).

## 4. Discussion

PR is an important economic swine disease caused by PRV that is difficult to eradicate in swine herds once infected. Since 2011, large-scale outbreaks of PR caused by PRV variants have appeared in China, which has brought catastrophic economic losses to the swine industry and simultaneously created difficulties in the control and prevention of PR (8). It is worth noting that PRV infection poses a health threat to humans. Therefore, it is of great significance to develop safe and effective drugs for PRV. Dandelion is a perennial herb of Asteraceae and has a long history as a Chinese herbal medicine to treat diseases, it contains numerous diverse bioactive compounds, such as triterpenoids, phenolic acids, polysaccharides, and flavonoids, which play an important role in antiviral, anti-inflammatory and antibacterial (22, 23). Additionally, there are relatively few side effects associated with dandelion, dandelion root, and dandelion extracts are authorized for use in dietary supplements by the FDA, with a “generally recognized as safe” status (34). In this study, we prepared an aqueous extract of dandelion to investigate its anti-PRV activity *in vitro* and *in vivo* and examined its mechanism of action against PRV.

The results obtained in this study indicated that DAE could inhibit PRV proliferation in PK15 cells in a concentration-dependent manner, and the SI value was 14.4 *in vitro*. By convention, tested drugs with an SI > 2 have low toxicity and are effective. The results suggested that DAE has a high potential value for utilization in the prevention of PRV infection.

The PRV life cycle mainly includes the stages of adsorption, entry, replication, assembly, and virus particle release (2). Each stage during the viral life cycle is critical and indispensable and can be considered a potential target of antiviral drugs. There have been many studies investigating the effects of drugs at different stages of PRV infection. Quercetin can prevent virus entry by interacting with the PRV gD protein (35). Glycyrrhiza polysaccharide inhibits PRV infection by blocking virus adsorption and internalization at the early stages of PRV infection (36). (-)-Epigallocatechin-3-gallate inhibits the adsorption, entry, and replication stages of PRV infection *in vitro* (37). The mode of action results in this study showed that DAE could not inhibit the cell entry stage of PRV infection but exerted an inhibitory effect by blocking PRV adsorption and replication in cells, which was similar to the mode of action of *Panax notoginseng* polysaccharides (38). The viral gene expression takes place after viral DNA reaches the cell nucleus and is the first step in viral replication. PRV infection is mainly achieved by controlling the level of expression, and blocking gene expression is an important way to inhibit viral proliferation (33). All tested genes (IE180, EP0, UL29, UL44, and UL52) in this study showed a reduction in expression levels after DAE treatments, confirming that DAE may block viral replication by inhibiting PRV gene expression. In addition, the gC protein encoded by the UL44 gene can mediate the binding of PRV to cell receptors, which is critical for PRV adsorption (2), and the ability of DAE to inhibit PRV adsorption to cells may be related to the suppression of UL44 gene expression.

PRV infects a wide host range, including a variety of mammals. Experimental animals (usually mice, rats, and rabbits) are highly susceptible to PRV infection under laboratory conditions. Mice and rats can be infected by different inoculation routes, and these animals are usually used as standardized models to study host responses to infection (7, 39). A mouse model was established by intramuscular injection in this study to explore whether DAE has antiviral effects against PRV infection *in vivo*. Additionally, the balance between the toxicity and antiviral activity of DAE is significant, and it should be ensured that the DAE dose has no effect on mouse health before experiments. *In vivo* tests showed that mice were healthy without any sign of toxicity by intraperitoneal injection of DAE at a dose of 0.5 g/kg body weight (unpublished results). Our results revealed that DAE treatment prolonged the mean survival time (MST) and increased the survival rate of mice. The viral load of the tested organs was detected by FQ-PCR, and the results showed that the viral load in the brain was the highest among these five organs, which might be related to the neurotropic nature of PRV. The addition of DAE significantly reduced the content of PRV in the brain, kidney, liver, lung, and heart of mice and efficiently inhibited PRV proliferation. According to the results of histopathological examination, the brains of mice infected with PRV showed pathological damage to varying degrees. Comparatively to the infected control group, only a small amount of lymphocyte infiltration was observed in the brain after DAE treatment, suggesting that DAE could alleviate histopathological damage induced by PRV infection. These results confirm that DAE could protect PRV-infected mice and has promising application prospects, and further tests can be conducted on pigs to better evaluate the anti-PRV activity of DAE.

The host's inflammatory response is the first line of defense to prevent the spread of virus infection. Cytokines are regulators of the host inflammatory response and play a protective role in resisting pathogen invasion. IFN-γ can activate macrophages, promote the production of proinflammatory factors such as TNF-α, regulate immunity, and play a significant role in antiviral immune responses (40). Three days after PRV infection in mice, the levels of TNF-α and IFN-γ were significantly increased, indicating that PRV infection activated the innate immune response of mice. After DAE treatment, the production of IFN-γ and TNF-α was higher than that in the infected control, especially IFN-γ, suggesting that DAE might inhibit viral infection in the early stage of PRV infection by enhancing the inflammatory response. The immunoregulatory cytokine IL-4 is mainly secreted by Th2 cells and plays a central role in promoting the development of Th2 responses and suppressing Th1 responses as well as immune responses (41). The results showed that the level of IL-4 was elevated after PRV infection, and DAE treatment significantly reduced the upregulation of IL-4 production caused by PRV infection, illustrating that DAE could modulate the IL-4 level and restore the Th1/Th2 balance in PRV-infected mice. These results suggest that DAE not only has a direct antiviral effect but also exerts anti-PRV activity by regulating the function of the host immune system. This mechanism contributes to the fight against virus infection, making the body less susceptible to resistance to DAE.

## 5. Conclusion

In conclusion, DAE possessed robust inhibitory activity on PRV infection *in vitro* and *in vivo*. DAE effectively inhibited PRV infection in PK15 cells, blocked PRV adsorption and replication, and reduced PRV gene expression. In animal experiments, DAE protected PRV-infected mice, which improved the survival rate, inhibited virus propagation, reduced pathological tissue changes, and regulate cytokine production to fight against viral infection. These results show that DAE can be further used as a therapeutic agent to prevent PRV infection.

## Data availability statement

The original contributions presented in the study are included in the article/Supplementary material, further inquiries can be directed to the corresponding author.

## Ethics statement

The animal study was reviewed and approved by Laboratory Animal Ethics Committee of Northeast Agricultural University.

## Author contributions

XC wrote the manuscript. XC, YS, ZW, YX, ZR, and LF performed the experimental work. YZ designed the experiments and revised the manuscript. All authors contributed to the article and approved the submitted version.
