# Supplementary material for: Antiviral activity of dandelion aqueous extract against pseudorabies virus both in vitro and in vivo
Source: Front Vet Sci. 2023 Jan 9;9:1090398. doi: 10.3389/fvets.2022.1090398 (PMC9870063; doi:10.3389/fvets.2022.1090398)
Supplement: Supplementary file 1 [file Table_1.docx]

| **Table S1. Primer sequences for quantitative PCR** | |
| --- | --- |
| Name | Sequence (5’-3′) |
| PRV-F | GCCGAGTACGACCTCTGCC |
| PRV-R | CGAGACGAACAGCAGCCG |
| PRV-Probe | HEX-CCGCGTGCACCACGAAGCCT-BHQ1 |
| UL44-F | CGTCAGGAATCGCATCA |
| UL44-R | CGCGTCACGTTCACCAC |
| IE180-F | CGCTCCACCAACAACC |
| IE180-R | TCGTCCTCGTCCCAGA |
| UL29-F | AGAAGCCGCACGCCATCACC |
| UL29-R | GGGAACCCGCAGACGGACAA |
| EP0-F | GGGCGTGGGTGTTT |
| EP0-R | GCTTTATGGGCAGGT |
| UL52-F | AGAAGCCGCACGCCATCACC |
| UL52-R | GGGAACCCGCAGACGGACAA |
| β-actin-F | TGCGGGACATCAAGGAGAA |
| β-actin-R | AGGAAGGAGGGCTGGAAGA |
